# Effect of *Citrus aurantium* L. Essential Oil on *Streptococcus mutans* Growth, Biofilm Formation and Virulent Genes Expression

**DOI:** 10.3390/antibiotics10010054

**Published:** 2021-01-08

**Authors:** Chahrazed Benzaid, Amine Belmadani, Lazhari Tichati, Ryad Djeribi, Mahmoud Rouabhia

**Affiliations:** 1 Oral Ecology Research Group, Faculty of Dentistry, Laval University, 2420, Rue de la Terrasse, Quebec City, QC G1V 0A6, Canada; chahrazed.benzaid@univ-annaba.dz (C.B.); amine.belmadani.1@ulaval.ca (A.B.); 2 Groupe de Recherche sur les Biofilms et la Biocontamination des Matériaux, Faculté des Sciences, Université d’Annaba, Annaba 23000, Algeria; rdjeribi@yahoo.fr; 3 Laboratoire de Bio Surveillance Environnementale, Université Badji Mokhtar, Annaba 23220, Algeria; lazhari.tichat@univ-annaba.org

**Keywords:** essential oil, *Citrus aurantium* L., *S. mutans*, caries, biofilms, gingival cells

## Abstract

In an oral cavity, dental caries, periodontal disease, and endodontic lesions are caused by well-known bacterial and fungal pathogens. Essential oils (EOs) have demonstrated antimicrobial activity suggesting their use for oral hygiene. The goal of this study was to evaluate the interaction of bitter orange flower (*Citrus aurantium* L.) essential oil with cariogenic bacteria *Streptococcus mutans* and human gingival epithelial cells. After extraction, the chemical composition of the essential oil was analyzed by gas chromatography, and its antimicrobial activity was evaluated against the growth and the expression of virulent genes in *S. mutans*. Finally, the effects of this essential oil on human gingival epithelial cell adhesion and growth were assessed using cell adhesion and proliferation assays. We showed that the major constituents of the tested essential oil were limonene, linalool, and β-ocimene. The essential oil reduced the growth of *S. mutans*, and decreased expression of *comC, comD, comE*, *gtfB, gtfC*, and *gbpB* genes. It should, however, be noted that essential oil at high concentration was toxic to gingival epithelial cells. Overall, this study suggests that *C. aurantium* L. essential oil could be used to prevent/control oral infections.

## 1. Introduction

Essential oils (EOs) are natural products empirically used to treat various illnesses [1,2]. In the literature on the subject, EOs have been suggested for various applications to treat sleep quality and anxiety as well as for antimicrobial products to synergize the antimicrobial activity of conventional antimicrobial agents [3,4]. In the oral cavity, the antimicrobial properties of EOs have shown promising health benefits, including the reduction of gingival inflammation and halitosis and the control of biofilm formation [5,6].

Biofilms refer to bacteria embedded with extracellular polysaccharides, lipoprotein, and fibrinogen. Biofilm formation is promoted by microorganism adherence to a favorable support and by their capacity producing extracellular matrix [7,8]. During biofilm formation, microorganisms modify their gene expression patterns. As an example, in *Streptococcus mutans,* genes associated with glucan (*gtfB*) and fructan synthesis (*ftf*) were regulate differentially in biofilm [9]. In *Candida albicans* biofilm formation involves different genes, including inositol phosphoryl transferase1 (*IPT1*) and *ECM33* genes [10,11]. It has been shown that biofilms confer a high bacterial resistance to antibiotics [12] leading to biofilm-related infections such as in oral cavities.

Oral microbiota is a normal component of an oral cavity and has an important function to protect against the colonization of extrinsic bacteria that could affect systemic health [13,14]. However, under certain conditions, including poor oral hygiene and the use of immunosuppressive drugs, commensal oral microbes can cause oral diseases such as dental caries and periodontal diseases [15], hence the emergence of multiple initiatives based on a modulation of the oral microbiome to maintain or reestablish a healthy oral ecosystem [16].

Oral hygiene is among the most efficient methods to prevent oral diseases [17]. This includes the use of mouthwash [18]. Mouthwash has become the ideal vehicle in which to incorporate antimicrobial molecules, as this easy-to-use product contributes to reducing plaque biofilm and preventing bad breath, to name a few [19]. Aside from antimicrobial peptides, mouthwash can also contain chlorhexidine, cetylpyridinium chloride, and essential oils [6]. These types of mouthwash can be considered therapeutic because they contain active ingredients intended to help control or reduce such conditions as halitosis, gingivitis, dental plaque, periodontitis, and tooth decay [19,20]. In the EOs used in mouthwash can be found thymol, menthol, and eucalyptol [21].

The EO extracted from *Citrus aurantium* L. (CA) has been reported to be efficient against microbial growth [22] and can serve as a food-flavoring substance [23,24] because it contains bioactive chemicals, such as phenolics and flavonoids [24,25]. With these reported beneficial effects, we sought to investigate the effects of the EO of CA on the oral bacteria *S. mutans* and to evaluate the safety/toxicity of this EO when in contact with gingival epithelial cells.

## 2. Results

### 2.1. Chemical Composition and Antioxidant Activities of C. aurantium L. Essential Oil

Chromatographic analyses resulted in the identification of 24 chemicals in the EO of CA (Table 1). The high-level chemicals in the EO were limonene (50.5%), linalool (9.89%), β-ocimene (7.82%), and sabinene (5.1%).

### 2.2. C. aurantium L. Essential Oil decreased S. mutans Cell Growth

**Figure 1:**
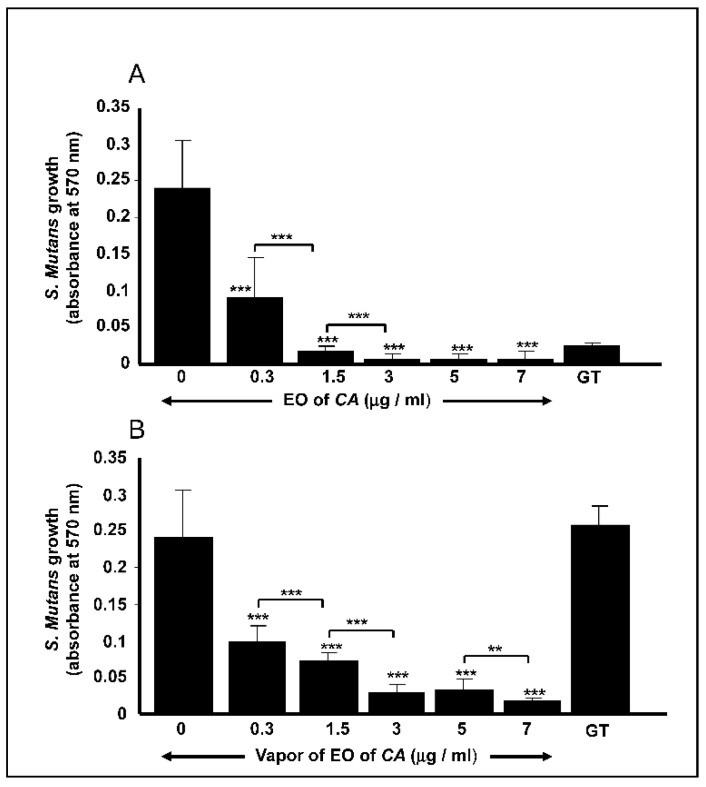
*Citrus aurantium* L. essential oil decreased *Streptococcus mutans* cell growth. Cell growth was evaluated after 24 h of either direct contact with the essential oil (EO) (**A**) or its vapor (**B**). Cell growth was assessed using crystal violet staining. GT = gentamicin. ** *p* < 0.01, *** *p* < 0.001.

Direct contact of *S. mutans* cells with the EO led to a significant (*p* < 0.01) inhibition of the bacteria growth (Figure 1A). After 24 h of culture, the inhibition of *S. mutans* cell growth was observed even at a low EO concentration. At 0.3 μg/mL, the OD_600_ decreased to 0.09 ± 0.05, compared to that recorded by the control, which was OD_600_ of 0.24 ± 0.067. At the highest concentration (7 μg/mL) of EO, the OD_600_ dramatically decreased to 0.009 ± 0.008, confirming the antimicrobial effect of the EO. It is interesting to note that starting from 1.5 μg/mL, the effect of the EO was greater than that of gentamicin at 5 μg/mL (Figure 1A). EO vapor was also found to be effective against *S. mutans* cell growth (Figure 1B). Indeed, with the lowest concentration (0.3 μg/mL), the EO vapor was capable of reducing *S. mutans* cell growth from 0.24 ± 0.067 (control) to 0.1 ± 0.02, which were comparable to the values obtained in the direct contact tests. At the highest concentration (7 μg/mL) of EO, the vapor produced an OD_600_ of 0.018 ± 0.005. Overall findings indicate that both direct contact and vapor contributed to significantly reducing the growth of the *S. mutans* cells.

### 2.3. C. aurantium L. Essential Oil Disrupted Mature S. mutans Biofilms

**Figure 2:**
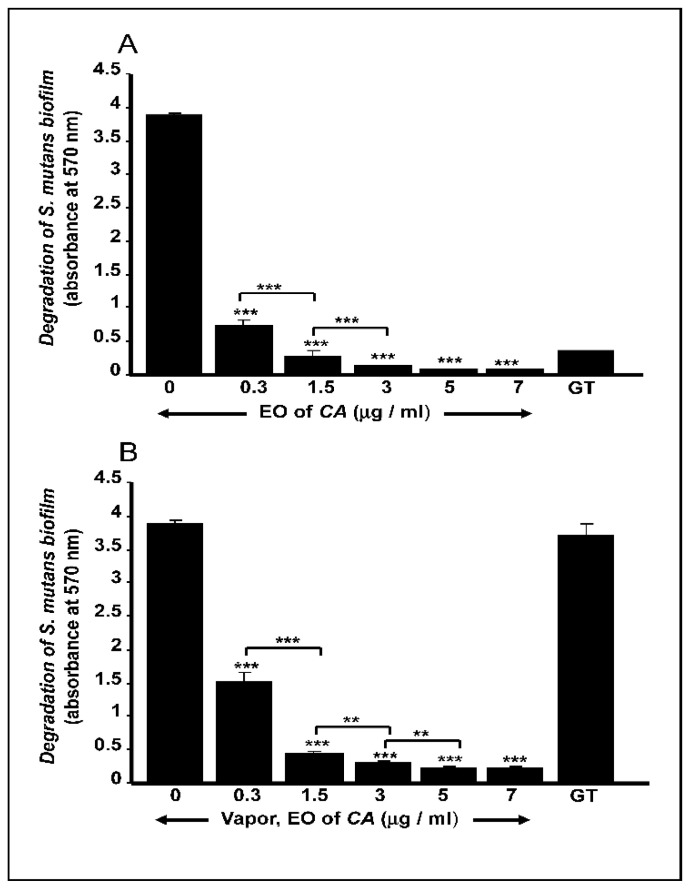
*C. aurantium* L. essential oil disrupted mature *S. mutans* biofilms. Bacteria were seeded into a 3D porous matrix for four days and then exposed to different concentrations of EO either in direct contact (**A**) or as vapor (**B**). Biofilm disruption was assessed by crystal violet staining. GT = gentamicin. ** *p* < 0.01; *** *p* < 0.001.

The mature biofilms showed a significant reduction in OD_570_ values following treatment with the EO (Figure 2). The presence of direct-contact EO was shown to degrade the biofilms, regardless of the concentration used (Figure 2A). The EO vapor also decreased *S. mutans* viability in the biofilms (Figure 2B). It is worth to be noted that both the direct and indirect contact with the EO resulted in a degradation of mature *S. mutans* biofilm in a dose-dependent manner (Figure 2). The effect of the EO on biofilm degradation was either equivalent to or greater (with 3 μg/mL of EO) than the effect obtained with gentamicin.

### 2.4. C. aurantium L. Essential Oil Decreased the mRNA Expression of Various Virulent Genes by S. mutans

**Figure 3:**
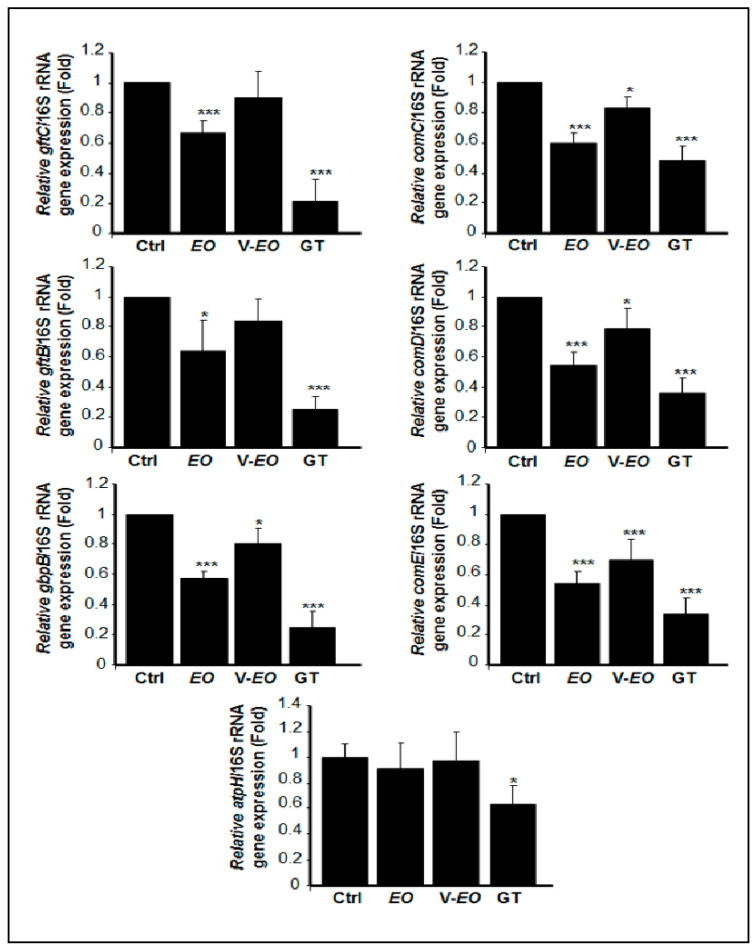
*C. aurantium* L. essential oil inhibited the expression of virulent genes by *S. mutans* cells. After exposure or not to the EO for 12 h, the total RNA was extracted and subjected to qPCR analyses. V-EO = essential oil vapor; GT = gentamicin; Ctrl = control. * *p* < 0.05; *** *p* < 0.001.

As shown in Figure 3, the mRNA expression of the *comC, comD, comE*, *gtfB, gtfC,* and *gbpB* genes significantly (*p* < 0.05) decreased following direct contact with the EO. Similar findings were observed with the EO vapor. However, the mRNA expression of *gtfB* and *gtfC* showed no significant change following the exposure of the *S. mutans* cells to the EO. Furthermore, the EO in both direct and indirect contact had no effect on the mRNA expression of *atpH* gene (Figure 3). It should be noted that the effect of gentamicin on all of the tested genes was higher than that obtained with the EO in either direct or indirect contact, although the decrease by the EO on gene expression remained significant.

### 2.5. C. aurantium L. Essential Oil Reduced Gingival Epithelial Cell Attachment

**Figure 4:**
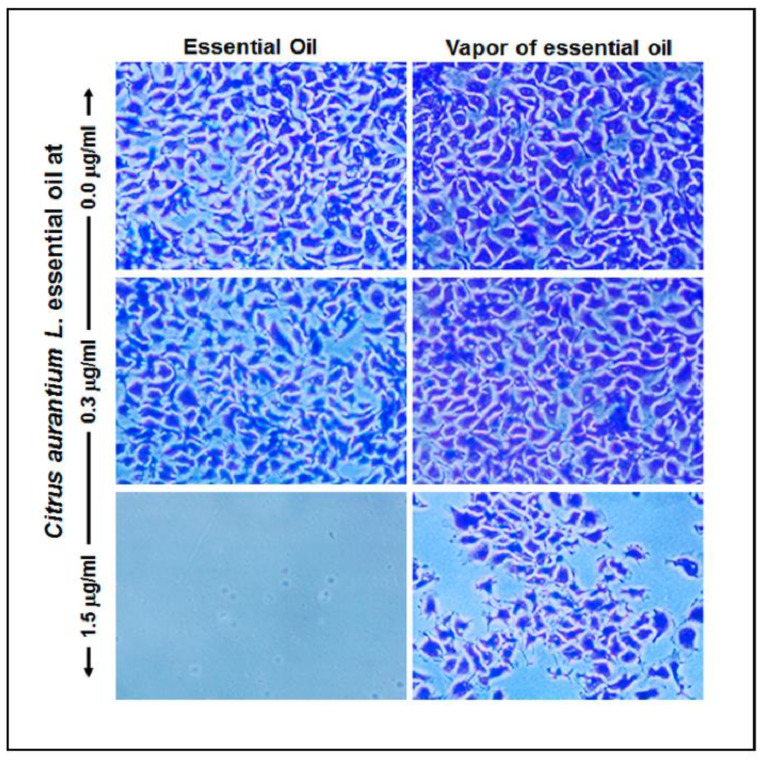
High concentrations of *C. aurantium* L. essential oil inhibited human gingival epithelial cell adhesion. Cells were seeded and exposed immediately to the EO for cell adhesion assessment. After 24 h incubation, the adherent cells were stained with crystal violet, cell adhesion was evaluated under an optical microscope and photographed.

**Figure 5:**
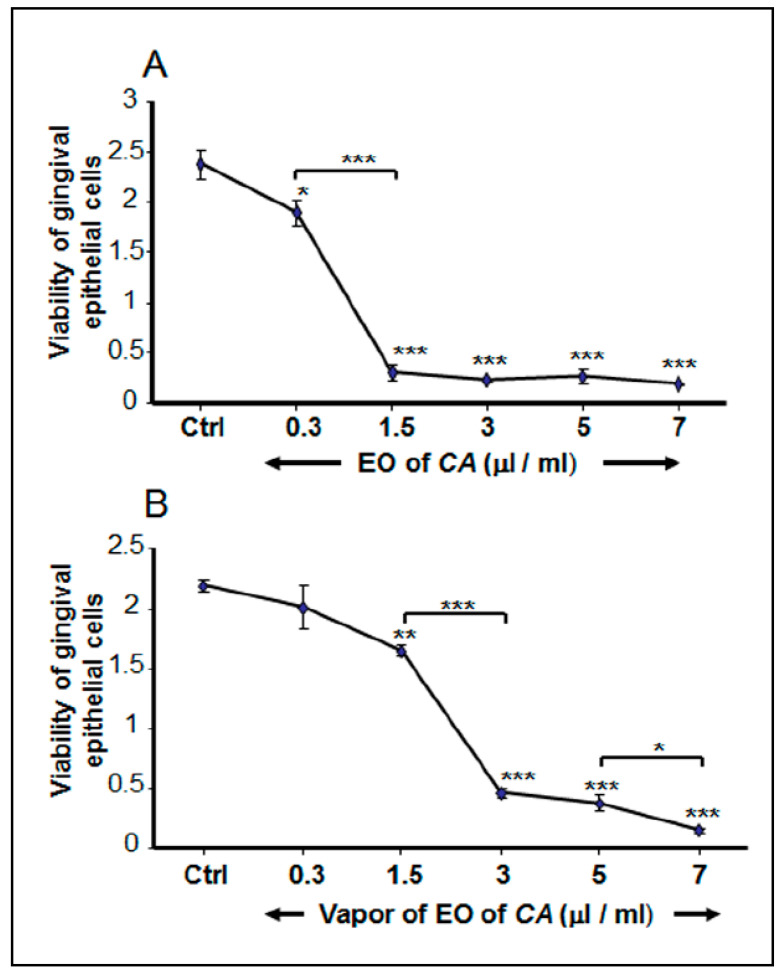
High concentrations of *C. aurantium* L. essential oil reduced human gingival epithelial cell viability. Cells were seeded and grow for 48 h prior to their exposure to the EO (**Panel A**) or the EO vapor (**Panel B**). After 48 h, the culture medium was refreshed and supplemented with various concentrations of the EO, and the cells were incubated for 24 h. Cell viability was assessed by MTT assay. * *p* < 0.05; ** *p* < 0.01, *** *p* < 0.001.

As shown in Figure 4 the addition of EO to gingival epithelial cell cultures had an important effect on the adhesion of these cells to the surface of the culture plates. Indeed, after 24 h of incubation, we observed the attachment of a high number of cells in the control groups and those supplemented with 0.3 μg/mL of the EO. In contrast, those fed with 1.5 μg/mL of the EO in direct contact resulted in total inhibition of cell adhesion. The EO vapor also had a significant reduction effect on gingival epithelial cell adhesion. While the cell cultures supplemented with a low concentration (0.3 μg/mL) of the EO showed good cell adhesion, at higher concentrations, cell adhesion was significantly inhibited (Figure 5). This suggests the use of low concentrations of the EO to prevent cell damage.

### 2.6. C. aurantium L. Essential Oil Decreased Gingival Epithelial Cell Viability

After 48 h of incubation, gingival epithelial cell cultures were supplemented or not with various concentrations of the EO through direct or indirect contact. The results presented in Figure 5A show that direct and indirect contact with the EO reduced cell viability. The MTT 3-(4,5-dimethylthiazol-2-yl)-2,5-monotetrazolium bromide assay (MTT) results reveal that with direct contact, the control cultures had a mean OD_550_ of 2.38 ± 0.14, and with the lowest concentration (0.3 μg/mL) of the EO, the OD_550_ dropped slightly, but significantly (*p* < 0.05) reaching to 1.9 ± 0.13. With the highest concentration (7 μg/mL) of the EO, epithelial cell viability dropped to an OD_550_ of 0.18 ± 0.01. Similar results were obtained with the EO vapor. As shown in Figure 5B, the viability of the epithelial cells went from 2.19 ± 0.05 with the control to 2.01 ± 0.18 with the lowest concentration (0.3 μg/mL) of the EO. This decrease was not significant as compared to the control (non-exposed cells). At high concentration (7 μg/mL), the OD_550_ dropped significantly (*p* < 0.01) reaching an OD_550_ of 0.43 ± 0.05. Overall results indicate that the use of EO vapor is less toxic to gingival epithelial cells.

## 3. Discussion

Combating the growth and virulence of oral pathogens, such as *C. albicans*, Porphyromonas *gingivalis*, *S. mutans*, etc., is important to ensure oral health. Frequent brushing with toothpaste reportedly reduces periodontitis and dental caries [26]. Furthermore, the use of cleaning solutions/mouthwash contributes to maintaining good oral hygiene by removing oral pathogens in dental plaque [27].

Mouthwashes contain various chemicals such as chlorhexidine, fluoride, delmopinol chloride, cetylpyridinium chloride, and alcohol, to name a few [28]. Although these ingredients are effective in reducing oral diseases, patients are concerned by the frequent use of these chemicals and are, therefore, looking for a mouthwash with natural products [29]. In this respect, essential oils may represent an interesting approach in the design of effective mouthwashes.

We extracted the essential oil from *C. aurantium* L. and analyzed its biological properties. The extracted EO had an elevated amount of limonene, followed by linalool. Both of these products display several attributes, including anti-inflammatory, antioxidant, anti-stress, and possibly disease-preventing properties [30,31]. This antioxidant activity is known to prevent tissue damage due to oxidation involving reactive oxygen species [32]. The antioxidant activity of the EO may be attributed to the presence of cineol, pinene, and limonene [33,34]. This activity was also in the range of what has been previously reported [22,33].

Limonene and linalool were shown to display antimicrobial properties [34], which may explain our results showing a significant decrease of *S. mutans* growth after exposure to the *C. aurantium* L. EO. These observations support those reported previously against *Listeria monocytogenes* [35] and aspergillus flavors [36], confirming the possible use of *C. aurantium* L. EO to control the growth of various bacterial strains. Growth inhibition may translate to reducing biofilm formation and disruption [37]. With this study, we are the first to show that *C. aurantium* L. EO can disrupt mature *S. mutans* biofilms.

The effects of the EO on *S. mutans* cell growth and biofilm degradation may involve certain virulent genes. Our results show that the EO is capable of decreasing *comC, comD, comE*, *gftB, gftC,* and *gbpB* mRNA expression in *S. mutans*. Activation of the *gtf* gene is known to lead to the production of GTFase synthesized glucans, which contribute to bacterial adherence and biofilm formation, while activation of the gtfB gene leads to the synthetization of polysaccharide containing α-1,3-linked glucans, facilitating cell aggregation in stable biofilms. The *gtfC* gene encodes the GTFC protein, which is involved in the synthesis of α-1,3- and α-1,6-linked glucans required for the formation and stabilization of biofilms [38]. The *gbpB* gene is involved in oral bacterial cell–cell aggregation, which facilitates the adhesion of the bacterium and the formation of mature biofilms [39].

*S. mutans* cell–cell interactions to form biofilm involve different gene products encoded by *comAB* [40] and *comCDE* [41]. The *comC*, *comD*, and *comE* genes, respectively, encode a competence-stimulating peptide (CSP) precursor contributing to biofilm formation [42]. In our study, the observed decrease in the mRNA expression of *comC, comD, comE*, *gftB, gftC,* and *gbpB* by the EO may explain the observed growth reduction and biofilm disruption.

The use of essential oil to prevent and/or control oral pathogens brings it into contact with other constituents in the oral cavity, such as the oral mucosa. For this reason, EO must be non-toxic to the host’s oral tissues. Our findings demonstrate that *C. aurantium* L. EO at 1.5 μg/mL and more is indeed toxic to gingival epithelial cells by inhibiting their adhesion and viability/growth. Of interest is that a low concentration (0.3 μg/mL) of EO showed reduced toxicity to the gingival epithelial cells, while it decreased significantly the growth of *S. mutans*, which supports the results of previous studies using various cell types [42].

The contribution of *C. aurantium* L. EO as a mouthwash supplement thus shows enormous potential, as its contact with human oral mucosa will only be for a short period (less than 30 s), and the oral mucosa tissue is more resistant (than are monolayer cell cultures). Furthermore, as shown in this study, even at a low concentration, *C. aurantium* L. essential oil is effective in reducing bacterial growth and degrading mature biofilms.

## 4. Materials and Methods

### 4.1. Plant Material, Extraction of C. aurantium L. Essential Oil, and Chemical Analyses

Bitter orange flowers (*C. aurantium* L.) (CA) were sampled during April 2016 from the Chiffa area in the central north region of Algeria. Botanical identification of the plant was determined in the Botany Laboratory at the Department of Biology at Badji Mokhtar—Annaba University. The extraction of the EO was carried out on fresh flowers using water vapor on an industrial scale by the Extral-Bio^®^ company located in Chiffa (Blida, Algeria). The extracted oil was subjected to gas chromatography (GC) analyses. Its components were characterized by comparing the retention indices on polar and non-polar columns using mass spectra compared with standard solutions of n-alkanes (C8–C28) as the control [43]. The GC conditions involved an injector with a temperature of 250 °C and the following injection mode: Split 1/50, volume injected: 0.2 mL. The column used was an HP-5 (L 30 m, internal diameter 0.25 mm; film thickness 0.25 mm) (Société Chromopic, Villejust, France). The stationary phase consisted of 5% phenyl and 95% dimethylpolysiloxane. The oven temperature was 60 °C for 8 min, followed by 2 °C/min up to 240 °C, and finally isothermal for 10 min. The duration of the analyses was 108 min and the carrier gas was nitrogen, with a flow of 0.5 mL/min.

### 4.2. Effect of C. aurantium L. EO on S. mutans Growth

*S. mutans* cells (ATCC 25175) were seeded (10^5^ CFU) in brain heart infusion (BHI) medium in the presence or absence of the EO at concentrations of either 0.3, 1.5, 3, 5, or 7 μg/mL. The EO concentrations were selected to identify the lowest concentration with antimicrobial activity and low toxicity for human cells. The EO was used in direct contact or as a vapor. For the vapor experiments, the EO was put into a sterile cotton swab and suspended over the surface of the culture medium at a distance of 3 cm (indirect contact). All the cultures were incubated aerobically at 37 °C in a 5% CO_2_ humid atmosphere. Negative (no EO) and positive (gentamicin (GT) at 5 μg/mL) controls were included in each experiment. Following incubation for 24 h, each culture was washed twice with Phosphate-buffered saline (PBS), after which time the pellets were suspended in 200 µL of a 1% crystal violet solution and incubated thereafter for 15 min under shaking conditions at 22 °C. Each cell suspension was then repeatedly suspended in distilled water then centrifuged until no visible blue color leached from the cell pellets. At this time, each pellet was air-dried for 24 h at 40 °C, suspended in 500 μL of a 30% acetic acid solution, and incubated for 15 min at 22 °C under agitation. The supernatants were distributed into a 96-well plate at 100 μL per well to measure the absorbance at 570 nm. Results were reported as the means ± SD, n = 6.

### 4.3. Effect of C. aurantium L. EO on the Disruption of Mature Biofilm

To produce mature biofilms, *S. mutans* (10^6^ CFU) cells were seeded into a 3D porous collagen matrix (5 × 5 mm) [44], after which time the samples were cultured for four days in BHI medium containing 1% glucose. Following this culture period, the biofilms were treated or not with the EO (0.3, 1.5, 3, or 7 μg/mL) under either direct contact or indirect vapor. Gentamicin-treated biofilms (5 μg/mL) were used as positive controls. The biofilms with the EO were treated for 24 h and subjected to crystal violet staining, and the OD_570_ was measured.

### 4.4. Effect of C. aurantium L. EO on the Activation/Repression of Various S. mutans Genes

*S. mutans* cells were cultured in the presence or absence of a chosen concentration (3 μg/ml) of the EO (direct contact or vapor) at 37 °C for 12 h. Following the incubation period, the RNA was extracted from each sample, as we previously reported [44] and was used to perform quantitative reverse transcription-polymerase chain reactions (RT-PCR). The RNA (500 ng of each sample) was reverse transcribed into cDNA by means of the iScript cDNA synthesis kit (Bio-Rad Laboratoties Ltd., Saint-Laurant, Quebec, Canada) and was subsequently used for quantitative PCR (qPCR). Reactions were performed using a PCR supermix (Bio-Rad, Laboratoties Ltd., Saint-Laurant, Quebec, Canada; iQ SYBR Green supermix). Specific primers for *comC, comD, comE, gtfB, gtfC*, *gbpB,* and *atpH* (Table 2) were added to the reaction mix at a final concentration of 250 nmol/l. Reactions were performed in triplicate, as we previously described [44]. The specificity of each primer pair was determined by the presence of a single melting temperature peak. The *16S rRNA* gene was included as a reference for this study. The results were analyzed using the 2^−ΔΔCt^ (Livak) relative expression method. The selected time (12 h) and concentration (3 μg/mL) were to enable us to collect enough cells to extract useful amounts of RNA. Indeed, a higher concentration of *C. aurantium* L. EO will reduce considerably the growth of *C. ablicans*, thus making it impossible to extract enough RNA for our gene expression analyses.

### 4.5. Effect of C. aurantium L. EO on Gingival Epithelial Cell Adhesion and Morphology

A gingival epithelial cell line (Ca9-22) purchased from the Health Science Research Resources Bank (Osaka, Japan) was seeded (5 × 10^5^) in sterile tissue culture Petri dishes in RPMI-1640 medium supplemented with 10% fetal calf serum. The EO at concentrations of 0, 0.3, 1.5, 3, 5, or 7 μg/mL was introduced to the cell cultures either in direct contact or as vapor. The cultures were then incubated at 37 °C for 24 h. The cells were then fixed with 4% paraformaldehyde for 60 min, stained with crystal violet dye, examined under an optical microscope, and photographed (*n* = 5).

### 4.6. Effect of C. aurantium L. EO on Gingival Epithelial Cell Growth and Proliferation

Ca9-22 cells (10^5^) were cultured in RPMI-1640 medium supplemented with 10% fetal calf serum for 48 h prior to contact with the EO. Following incubation, the culture medium was refreshed and the cells were exposed to the EO (direct contact or vapor) and subsequently incubated for 24 h in a 5% CO_2_ humid atmosphere at 37 °C. An MTT assay was then performed on each culture, as we previously reported [45]. The results were presented as the means ± SD; (*n =* 5).

### 4.7. Statistical Analysis

Each experiment was performed at least four times, with experimental values expressed as means ± SD. The statistical significance of the differences between the control (absence of the EO) and test (presence of the EO or gentamicin) values was determined by means of a one-way ANOVA. Posteriori comparisons were conducted using Tukey’s method. *P* values were declared significant at ≤ 0.05. The data were analyzed using a statistical version 8.2 (SAS Institute Inc., Cary, NC, USA).

## 5. Conclusions

In this study, we demonstrate that *Citrus aurantium* L. essential oil could decrease the growth of *Streptococcus* *mutans* and degrading its mature biofilms. Our findings also show a significant decrease in certain virulent genes (*comC, comD, comE*, *gtfB, gtfC*, and *gbpB*) expressed by *S. mutans*. While at mid and high concentrations, the essential oil (EO) was toxic to gingival epithelial cells, this toxicity was lower with a low concentration of the EO, which was effective in reducing *S. mutans* growth. Overall results suggest the possible use of *C. aurantium* L. essential oil to reduce the pathogenesis of *S. mutans*.

## Figures and Tables

**Table 1:** Identification of the different chemicals in the *Citrus aurantium* L. Essential oil by means of gas chromatography (GC) analyses.

Compound	Retention Time (min)	Retention Index	Aire%
α-Pinene	3.22	936	0.63
Camphene	3.59	955	0.02
β-pinene	4.30	990	3.00
**Sabinene**	**4.59**	**1001**	**5.01**
β-Myrcene	5.17	1026	2.17
δ-3- carene	5.54	1048	0.86
**Limonene**	**5.62**	**1053**	**50.56**
**(E)-β—Ocimene**	**5.89**	**1063**	**7.82**
**Z- β—Ocimene**	**6.38**	**1075**	**3.54**
γ—Terpinene	6.59	1088	0.64
Terpinolene	6.77	1102	0.48
Citronellal	7.54	1139	0.27
**Linalool**	**7.71**	**1152**	**9.89**
Terpinen-4-ol	8.03	1175	0.32
Linalylacetate	8.39	1281	1.61
α—Terpineol	8.83	1185	0.12
Neral	12.163	1268	0.08
Geraniol	13.82	1280	1.53
Geranial	14.34	1291	0.26
Nerylacetate	19.73	1364	1.52
Geranylacetate	20.09	1385	2.26
Methyl N-methylanthranilate	22.17	1402	2.35
Betacaryophyllene	22.60	1416	1.21
**Total**			**96.15%**

**Table 2:** Primer sequences used for the quantitative polymerase chain reaction (qPCR).

Gene	Primer Sequence (5′ à 3′)	Annealing Temperature (°C)	Amp Size (bp)
*16S rRNA*	Forward: CTTACCAGGTCTTGACATCCCG Reverse: ACCCAACATCTCACGACACGAG	63.3	113
*gbpB*	Forward: AGCAACAGAAGCACAACCATCAG Reverse: CCACCATTACCCCAGTAGTTTCC	65.6	150
*gtfB*	Forward: ACACTTTCGGGTGGCTTG Reverse: GCTTAGATGTCACTTCGGTTG	62	127
*gtfC*	Forward: CCAAAATGGTATTATGGCTGTCG Reverse: GAGTCTCTATCAAAGTAACGCAGT	63	135
*comC*	Forward: GACTTTAAAGAAATTAAGACTG Reverse: AAGCTTGTGTAAAACTTCTGT	54	103
*comD*	Forward: CTCTGATTGACCATTCTTCTGG Reverse: CATTCTGAGTTTATGCCCCTC	62	150
*comE*	Forward: CCTGAAAAGGGCAATCACCAG Reverse: GGGGCATAAACTCAGAATGTGTCG	65	148
*atpH*	Forward: ACCATACATTTCAGGCTG Reverse: TTTTAGCACTTGGGATTG	56	101

## Data Availability

All data were included in the manuscript.
